# Nutrient Properties and Nuclear Magnetic Resonance-Based Metabonomic Analysis of Macrofungi

**DOI:** 10.3390/foods8090397

**Published:** 2019-09-07

**Authors:** Dan Liu, Yu-Qing Chen, Xiao-Wei Xiao, Ru-Ting Zhong, Cheng-Feng Yang, Bin Liu, Chao Zhao

**Affiliations:** 1 College of Food Science, Fujian Agriculture and Forestry University, Fuzhou 350002, China (D.L.) (Y.-Q.C.) (X.-W.X.) (R.-T.Z.) (B.L.); 2 College of Food Science and Nutritional Engineering, China Agricultural University, Beijing 100083, China; 3 China National Engineering Research Center of JUNCAO Technology, Fuzhou 350002, China; 4 Key Laboratory of Marine Biotechnology of Fujian Province, Institute of Oceanology, Fujian Agriculture and Forestry University, Fuzhou 350002, China

**Keywords:** macrofungi, proximate compositions, small molecules, metabonomics, NMR

## Abstract

Many delicious and nutritional macrofungi are widely distributed and used in East Asian regions, considered as edible and medicinal foods. In this study, 11 species of dried and fresh, edible and medicinal macrofungi, *Ganoderma amboinense*, *Agaricus subrufescens*, *Dictyophora indusiata*, *Pleurotus sajor-caju*, *Pleurotus ostreatus*, *Pleurotus geesteranu*, *Hericium erinaceus*, *Stropharia rugosoannulata*, *Pleurotus sapidus*, *Antrodia camphorata*, and *Lentinus edodes* (Berk.) Sing, were investigated to determine the content of their nutritional components, including proteins, fat, carbohydrates, trace minerals, coarse cellulose, vitamins, and amino acids. The amino acid patterns and similarity of macrofungi were distinguished through principal component analysis and hierarchical cluster analyses, respectively. A total of 103 metabolic small molecules of macrofungi were identified by nuclear magnetic resonance spectroscopy and were aggregated by heatmap. Moreover, the macrofungi were classified by principal component analysis based on these metabolites. The results show that carbohydrates and proteins are two main components, as well as the nutritional ingredients, that differ among various species and varied between fresh and dried macrofungi. The amino acid patterns in *L. edodes* and *A. subrufescens* were different compared with that of the other tested mushrooms.

## 1. Introduction

**Figure 1:**
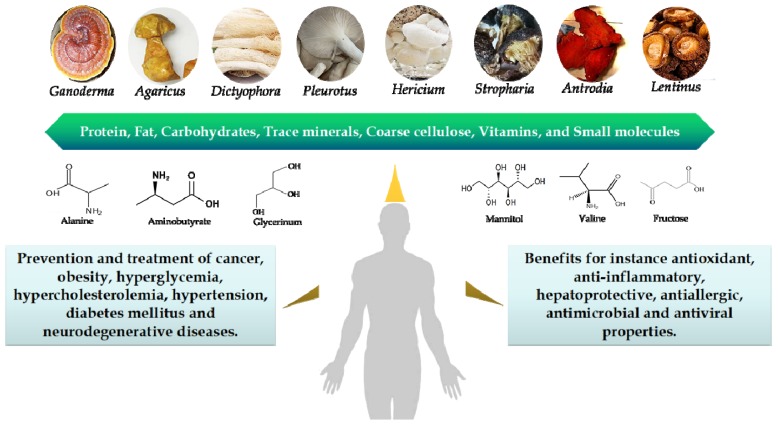
The beneficial roles of edible and medical mushrooms in human health.

More than 12,000 species of macrofungi (mushrooms) have been found, and there are at least 2200 edible species in the world [1]. Thousands of years ago, China, Japan, Korea, and parts of Africa have used macrofungi for nutritional and medical purposes. Edible mushrooms also are cultivated for consumption worldwide because of their special valued nutrients [2,3,4]. *Agaricus subrufescens*, *Dictyophora indusiata*, *Pleurotus sajor-caju*, *Lentinus edodes* (Berk.) Sing, *Pleurotus geesteranu*, *Stropharia rugosoannulata*, and *Pleurotus sapidus* offer special umami tastes, making them delicious table dishes. *A. subrufescens*, whose fruiting body is called “*Himematsutake*” in Japan, is regarded as a health food and is a significant component of traditional Chinese medicine. The so-called queen of mushrooms, *D. indusiata*, is famous for its good-looking appearance and delicious taste. Its polysaccharides have been verified to prevent tumor and oxidant stress [5,6]. Also, it has been demonstrated that glucan-rich polysaccharides from *P. sajor-caju* can prevent glucose intolerance and inflammation in high-fat diet mice [7]. The extracts of mycelium of *Pleurotus ostreatus* have been identified to be rich in selenium and to prevent cancer [8]. Moreover, the triterpene of *Ganoderma amboinense* has been documented to induce the senescence of HepG2 cells [9]. In addition, the extracts of *Hericium erinaceus* and *Antrodia camphorata* can manage neurodegenerative diseases, which has been utilized as Chinese medicine and in functional foods (Figure 1) [10]. In recent years, the nutritional values of edible mushrooms have attracted more and more attention globally [3,4,11]. There are many other biological activities, such as antifungal, antibacterial, immunomodulatory, antiviral [12], anti-inflammatory [13], cholesterol-reducing [14], anti-cytotoxic, and antihypoglycemic [3,11,15] effects that have been reported. The various functions of fungi are closely correlated with their abundant nutrients. The fruiting bodies of mushrooms are considered as the main sources of organic nutrients, which include abundant proteins, digestible and non-digestible carbohydrates, fiber, vitamins, mineral contents, and a low level of fat [16,17,18]. Edible mushrooms possess a large resource of proteins with abundant essential amino acids, with their ratio among total amino acids reaching from 30% to 40% [19]. The digestible carbohydrates contained mannitol (0.3%–5.5% dried sample weight (dw)) [20], glucose (0.5%–3.6% dw) [21], and glycogen. Non-digestible carbohydrates take up a large part of the total carbohydrates, and mainly include oligosaccharides and non-starch polysaccharides, such as chitin, crude fibre, β-glucans, and mannans [22].

In the last decade, the identification of mushrooms’ proximate compositions and small molecules has mostly been investigated. Nonetheless, the broad, large-scale comparative metabolomics studies were lacking in terms of edible and medical mushrooms. In this study, the systematic broad-scale metabolomics of 11 species of mushrooms are investigated, including *G. amboinense*, *A. subrufescens*, *D. indusiata*, *P. sajor-caju*, *P. ostreatus*, *P. geesteranu*, *H. erinaceus*, *S. rugosoannulata*, *P. sapidus*, *A. camphorata*, and *L. edodes* (Berk.) Sing. Nuclear magnetic resonance (NMR) spectroscopy, a well-developed technique to perform metabolite profiling, was used to detect different metabolites and their assigned substances involved in metabolic pathways. Major metabolic and components differences between selected mushrooms and close relatives were also demonstrated. There is little relative research systematically focused on the metabolomic analysis of these mushrooms.

## 2. Materials and Methods

### 2.1. Cultivated Edible and Medical Mushrooms

The fruiting bodies of *G. amboinense*, *A. subrufescens*, *D. indusiata*, *P. sajor-caju*, *P. ostreatus*, *P. geesteranu*, *H. erinaceus*, *S. rugosoannulata*, *P. sapidus*, and *L. edodes* (Berk.) Sing were grown and harvested in the mushroom farm at the China National Engineering Research Center of JUNCAO Technology (Fuzhou, China). *A. camphorata* was obtained from the Institute of Food Science and Technology, National Taiwan University. The mushrooms were cut into pieces and were dried in an oven at 65 °C for 24 h, and then were grinded into powder by an ultrafine grinder (Hangzhou, China) for further detection. At the same time, the fresh mushrooms were smashed into particles with 2 mm diameter for comparison with the dried fungi. Then, the dried and fresh mushrooms were stored at −80 °C for further analysis.

### 2.2. Proximate Compositions and Trace Minerals

The chemical compositions (proteins, fat, and carbohydrates) of edible and medical mushrooms were analyzed by the Association of Official Analytical Chemists (AOAC) method [23]. The crude protein was estimated by Micro-Kjeldahl methods [24]. The crude fat was assessed through extracting a known weight of powdered sample with petroleum ether by Soxhlet apparatus. The carbohydrate contents of fungi were recalculated according to the glucose contents. The mineral elements of Cu, Fe, Zn, Mn, and Ca were detected by inductively coupled plasma-atomic emission spectrometry (ICP-AES) [24,25].

### 2.3. Coarse Fibers

The procedure of cellulose extraction was adjusted from the work done by Morán et al. [26]. The samples were washed several times by distilled water, dried by drying oven and cut to an approximate length of 6–12 mm. After that, the blocks were boiled with toluene/ethanol mixture (2:1, *v/v*) by Soxhlet apparatus for 6 h, then filtered and washed by ethanol for 30 min to be dried. The samples were incubated with 0.1 M NaOH (dissolved in 50% volume of ethanol) at 45 °C for 3 h under continuous agitation. The pre-treated samples were then treated with H_2_O_2_ at different concentrations (0.5%, 1.0%, 2.0%, and 3.0%, dissolved in buffer solution, pH 11.5) at 45 °C for 3 h under continuous agitation. Following that, they were incubated with 10% NaOH (*w/v*) and 1% Na_2_B_4_O_7_ (*w/v*) at 28 °C for 15 h, and then with 70% HNO_3_ and 80% HAc at 120 °C for 15 min. Finally, the extracts were washed by 95% ethanol, water, and 95% ethanol in turn, and dried at 60 °C in an oven until constant weight.

### 2.4. Vitamin A and C

Vitamin A content was analyzed by HPLC system with a fluorescence detector FP-2020 (Jasco, Tokyo, Japan) programmed at 320–330 nm [18,27]. The chromatography was used to identify the compounds by comparisons with standards (Sigma-Aldrich, Louis, MO, USA). On the basis of the fluorescence signal response of each standard, the internal standard method and calibration curves obtained were performed to quantify the content (μg) of vitamin A among 100 g dried sample weight (dw). The content of ascorbic acid (vitamin C) was measured by the 2,6-dichloroindophenol titrimetric method [28] on the basis of the calibration curve of L-ascorbic acid, in which the results were expressed as milligrams of ascorbic acid per 100 g of dw.

### 2.5. Amino Acids

Amino acid contents of samples were analyzed by gas chromatographic (GC) method as the previous study described [29]. Ten grams of each mushroom sample was defatted through extraction with 30 mL of the petroleum spirit three times by Soxhlet apparatus. The defatted samples were then hydrolyzed thoroughly at 112 °C for 24 h three times. The protein hydrolysates were extracted with 30 mL of dichloromethane three times until the final volume was 1.0 mL, then a GC system with a mass selective detector was used to test the amino acid content of the mushroom samples.

### 2.6. Sample Preparation and ^1^H NMR Spectroscopic Measurement

The samples were defrosted at room temperature, and 250 μL aliquots were mixed with 250 μL of 1.5 M phosphate buffer (pH 7.4) to minimize variation in pH. The samples were centrifuged at 13,000 rpm for 10 min at 4 °C to separate any precipitate. Then, the filter liquor was added with 4, 4-dimethyl-4-silapentane-1-sulfonic acid (DSS) (50 μL) and vortexed for 10 s. Afterwards, the mixed liquor was centrifuged for 2 min at 13,000 rpm. Finally, the 480 μL of total supernatant was injected into the nuclear magnetic tube. ^1^H NMR was measured on a DD2 600 MHz spectrometer (Agilent, CA, USA) operating at a 599.83 MHz magnet frequency and equipped with a triple-resonance cryoprobe. Therein, 256 scans were collected with a spectral width of 7225.434 Hz at 25 °C; the recycle delay time was set as 0.01 s and the water signals were suppressed during relaxation time. The free induction decay (FID) was transported into the Chenomx NMR suit (version 8.0, Edmonton, Alberta, Canada) software, and the ^1^H NMR spectra were manually phased and baseline-corrected. The DSS at 0.0 ppm was used as reference for chemical shifts.

### 2.7. Statistical Analysis

For this study, data of amino acid derivatives and peptides, as well as metabolic profiling, were analyzed by unsupervised principle component analyses (PCA) and hierarchical cluster analyses (with complete linkage) using SIMCA-14.1 software (UMETRICS, Umea, Sweden) based on the Ward algorithm. For PCA, the data of mushrooms’ components were normalized after filling the baseline value. Briefly, the logarithm of variance was calculated, and the Pareto scaling method was used for scaling. Then, a similar method was used in clustering analysis based on the results of PCA. The quantity and quality of metabolites were analyzed by Chenomx NMR suite (Version 8.0, Edmonton, Alberta, Canada). The relative abundances of small molecules from dried and fresh mushrooms were further visualized with heatmap by utilizing the R-3.2.2 software (Auckland University, Auckland, New Zealand) with a heatmap package.

## 3. Results and Discussion

### 3.1. Proximate Compositions and Trace Elements from Dried and Fresh Mushrooms

**Figure 2:**
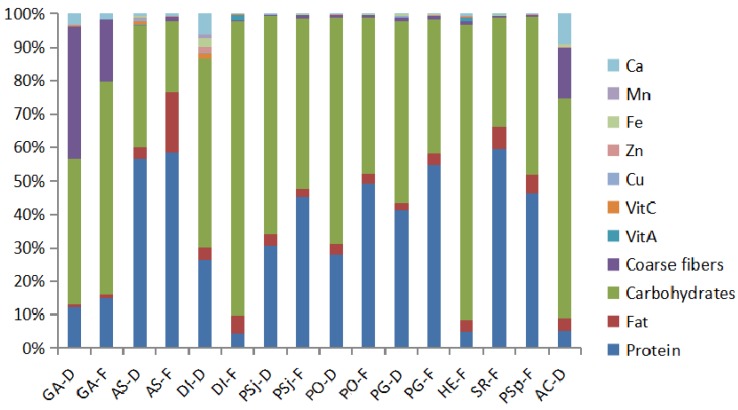
Proximate compositions and trace elements from the dried and fresh mushrooms. Note: GA-D, *G. amboinense* (dried); GA-F, *G. amboinense* (fresh); AS-D, *A. subrufescens* (dried); AS-F, *A. subrufescens* (fresh); DI-D, *D. indusiata* (dried); DI-F, *D. indusiata* (fresh); PSj-D, *P. sajorcaju* (dried); PSj-F, *P. sajorcaju* (fresh); PO-D, *P. ostreatus* (dried); PO-F, *P. ostreatus* (fresh); PG-D, *P. geesteranu* (dried); PG-F, *P. geesteranu* (fresh); HE-F, *H. erinaceus* (fresh); SR-F: *S. rugosoannulata* (fresh); PSp-F, *P. sapidus* (fresh); AC-D, *A. camphorata* (dried).

Edible mushrooms contain various kinds of nutrients, such as protein, fat, carbohydrates, and vitamins, which are the most essential macronutrients in human life with comprehensive nutritional values. Proximate compositions and trace elements from dried and fresh edible mushrooms were determined as shown in Figure 2 and Table 1. Eleven edible and medical mushrooms could be classified as follows: Agaricales (*A. subrufescens*, *P. sajor-caju*, *P. ostreatus*, *P. geesteranu*, *S. rugosoannulata*, *P. sapidus*, *L. edodes* (Berk.) Sing), Aphyllophorales (*G. amboinense*, *A. camphorata*), Phallales (*D. indusiata*), and Russulales (*H. erinaceus*). The content of nutrients varied in different species of fresh or dried mushrooms. Moreover, there was a vast difference in nutritional compositions between DI-F (*D. indusiata* (fresh)) and DI-D (*D. indusiata* (dried)), or PG-F (*P. geesteranu* (fresh)) and PG-D (*P. geesteranu* (dried)) (Table 1). The level of fat content was low in all mushrooms, except fresh *A. subrufescens* (15.7 g/100 g), which was similar to previous research, such as in the content of crude fat being 10.7 g/100 g in *Agricus* as reported by Liu et al. (2019) [30,31]. The protein and carbohydrates are the two main compounds of mushrooms, and the content of protein in Agricales mushrooms was generally higher than other mushrooms (Figure 2), with the highest content being found in fresh *A. subrufescens* (50.4 g/100 g) (Table 1). However, the levels of carbohydrates in Aphyllophorales, Phallales, and Russulales were higher than Agricales mushrooms. The total fibre of Aphyllophorales was also found to be higher than other, and the highest level of total fiber (40.8%) was presented in dried *G. subrufescens*. Dried *A. subrufescens* had the lowest (0.075%) content of total fiber, which was same as previous research results [32,33]. Above all, the rich amount of proteins, essential amino acids, carbohydrates, and essential minerals, in contrast to low fat levels, make many mushrooms a good choice for consumers. Vitamins and mineral elements play an indispensable role in satisfying bodily demand to promote health. *D. indusiata* and *H. erinaceus* showed the highest contents of vitamin A, with 770 μg/100 g and 550 μg/100 g, respectively. However, it was not detected in *A. camphorata*. Dried *D. indusiata* contained the highest level of vitamin C (111.4 mg/100g), and dried *A. subrufescens* (69.7 mg/100 g) followed. The trace elements, especially calcium, were abundantly found in Aphyllophorales and Phallales mushrooms (Figure 2). Dried *D. indusiata* had the highest contents of Zn (130 mg/kg), Fe (184 mg/kg), and Mn (75 mg/kg), which is consistent with previous research [34]. The highest levels of Cu and Ca were found in dried *A. subrufescens* and *A. camphorata*, respectively. Furthermore, dried and fresh samples had nearly the same composition. HE-F (*H. erinaceus* (fresh)) had the same composition with that in AS-F (*A. subrufescens* (fresh)), but the content of carbohydrate in HE-F was more than twice that of AS-F, while the protein content was twice more than that in GA-F (*G. amboinense* (fresh)).

### 3.2. Amino Acid Derivatives and Peptides

**Figure 3:**
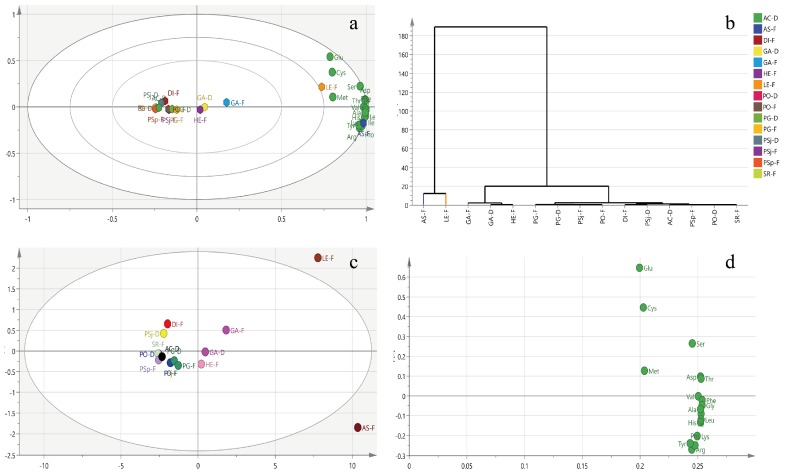
Biplot (**a**), clustering analysis (**b**), score plot (**c**), and loading plot (**d**) of amino acids in dried and fresh edible mushrooms. Note: GA-D, *G. amboinense* (dried); GA-F, *G. amboinense* (fresh); AS-D, *A. subrufescens* (dried); AS-F, *A. subrufescens* (fresh); DI-D, *D. indusiata* (dried); DI-F, *D. indusiata* (fresh); PSj-D, *P. sajorcaju* (dried); PSj-F, *P. sajorcaju* (fresh); PO-D, *P. ostreatus* (dried); PO-F, *P. ostreatus* (fresh); PG-D, *P. geesteranu* (dried); PG-F, *P. geesteranu* (fresh); HE-F, *H. erinaceus* (fresh); SR-F: *S. rugosoannulata* (fresh); PS-F, *P. sapidus* (fresh); AC-D, *A. camphorata* (dried); LE-F, *L. edodes* (Berk.) Sing (fresh).

Amino acids can be divided into essential (eight kinds of amino acids for adults and nine kinds for infants), non-essential, and conditionally essential amino acids (cysteine (Cys) and tyrosine (Tyr)). The amino acids in these selected edible and medical mushrooms were of high quality and substantial similarity. The mushrooms mostly contained nine essential, six non-essential, and one kind of conditionally essential amino acids. To some extent, they can virtually be substituted for meat, eggs, and milk [19]. However, the contents of amino acids in fresh mushrooms were higher compared with dried samples, and different types of amino acids were varied in those selected mushrooms (Figure 3a and Table 2). The highest levels of total essential and conditionally essential amino acids were both found in fresh *A. blazei* at 133.7 and 5.9 g/kg, respectively. Total non-essential amino acids were found in *L. edodes* at 110.1 g/kg, which was slightly less than that of 144 g/kg tested by Li et al. (2018) [35]. *A. subrufescens* had the highest essential amino acid content, except for methionine (Met). Moreover, other mushrooms were also found to be rich in essential amino acids, especially *L. edodes* and *G. subrufescens*. Glutamate (Glu) is a major component of non-essential amino acids, which exists largely in selected mushrooms. In addition, tyrosine (Tyr), as a conditionally essential amino acid, was distributed in all selected mushrooms, especially in *L. edodes* (4.8 g/kg), *A. subrufescens* (5.9 g/kg), and *G. subrufescens* (2.8 g/kg).

Amino acids of mushrooms were classified by principal component analysis (PCA) as an unsupervised multidimensional statistical analysis method. Meanwhile, the principal components were acquired based on the content of metabolites which were measured by NMR. The model of all samples explained 95.03% of the principal components, with principal component 1 (PC1) interpreting 90.9% and principal component 2 (PC2) interpreting 4.13% of the total variance. The biplot indicated that the fresh *L. edodes* (Berk.) Sing and fresh *A. subrufescens* were remarkably separate from other mushrooms according to PC1 (Figure 3a). GA-D (*G. amboinense* (dried)), HE-F, GA-F, LE-F (*L. edodes* (Berk.) Sing (fresh)), and AS-F were located at PC1 with positive scores, nevertheless other mushrooms presented negative scores on PC1, suggesting that LE-F and AS-F were completely dissimilar in amino acid patterns compared with other mushrooms. LE-F and AS-F were further segregated on the direction of PC2, indicating the amino acid profiles were also different between them. The nutrients compositions of AS-F and AE-F were different with that of the other mushrooms (Figure 3c). The higher absolute value of data in loading plot meant the bigger contribution to the principle compounds. The loading plot of amino acids showed that Glu, Cys, and Met were the most contributive principles discriminative of LE-F, while Val, Phe, Gly, Ala, His, Ile, Pro, Lys, and Arg of AS-F were different compared with other mushrooms (Figure 3d). Hierarchical cluster analysis, a method to quantify the similarity of different mushrooms, was carried out based on amino acid profiles. All samples could be classified into three clusters if the phenon line was defined as the distance of 20. Cluster I was composed of AS-F and LE-F. Cluster II was made up with GA-D, GA-F, and HE-F. Most mushrooms were gathered at Cluster III, which included PG-F, PG-D, PSj-F (*P. sajorcaju* (fresh)), PO-F (*P. ostreatus* (fresh)), DI-F, PSj-D (*P. sajorcaju* (dried)), AC-D (*A. camphorata* (dried)), PSp-F (*P. sapidus* (fresh)), PO-D (*P. ostreatus* (dried)), and SR-F **(***S. rugosoannulata* (fresh)) (Figure 3b).

### 3.3. Metabolic Profiling of Selected Mushrooms

A total of 103 different kinds of small molecules, including organic acids, amino acids, polyols, amines, sugars, vitamins, esters, and others were determined in the dried and fresh mushrooms by NMR (Table S1 and Figure S1). It was identified that the PG-D had the most content of metabolites, which was followed by fresh *P. sapidus* and dried *H. erinaceus*. 4-Aminobutyrate, formate, fumarate, glucose, glutamine, isoleucine, methanol, serine, sn-glycero-3-phosphocholine, uridine, and valine were found to be existing in all tested fungi, and the content of them in different fungi was diverse. The total content of trehalose in all mushrooms was the most prevalent, especially present in fresh *P. sajorcaju*, followed by mannitol (Table S1). Total glucose also made up a relatively significant share in all water-soluble small molecules, especially in DI-D, and the glucose content of *D. indusiata* was similar to the former study [36]. Mannitol existed in all selected mushrooms, which can support and expand the mushroom fruiting bodies [30].

**Figure 4:**
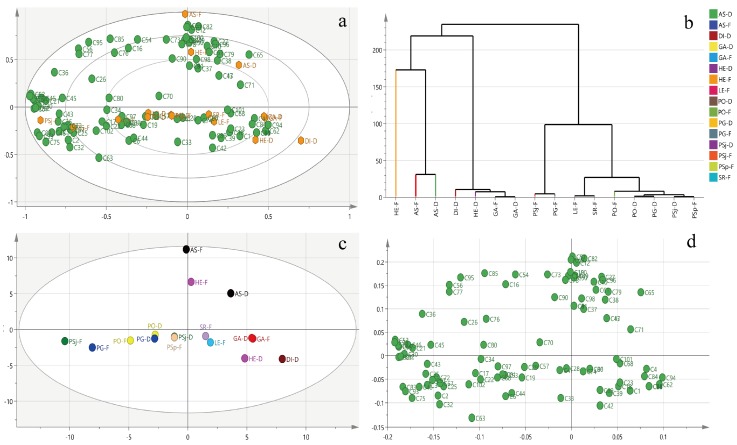
Biplot (**a**), clustering analysis (**b**), score plot (**c**), and loading plot (**d**) of metabolites.

Metabolites of edible and medical mushrooms were classified by PCA (Figure 4a). Meanwhile, the principal components were acquired based on the content of metabolites using NMR (Table S1). The model of all samples explained 41.4% of the principal components, with principal component 1 (PC1) interpreting 24.9% and principal component 2 (PC2) interpreting 16.5% of the total variance. The mushrooms were categorized into three clusters based on metabolites using the ward algorithm: Cluster Ⅰ (HE-F, AS-D (*A. subrufescens* (dried)), and AS-F), Cluster Ⅱ (PSj-F, PG-D, PSj-D, PO-D, and PO-F), and Cluster Ⅲ (SR-F, LE-F, GA-D, GA-F, HE-D (*H. erinaceus* (dried)) and DI-D) (Figure 4b). The loading plot of metabolites showed that different metabolites were distributed in different quadrants (Figure 4c,d). For example, uridine (C98), mannitol (C61), propylene glycol (C79), proline (C78), inosine (C50), and valine (C100) were the main important discriminative factors contributing to HE-F from other mushrooms. Hierarchical cluster analyses of different mushrooms based on metabolites showed that samples could be sorted into four clusters if the phenon line was defined as the distance of 100. Cluster I was composed of HE-F; Cluster II was made up with AS-F and AS-D; Cluster III contained DI-D, HE-D, GA-F, and GA-D. Most mushrooms were sorted on Cluster IV, which included PSj-F, PG-F, LE-F, SR-F, PO-F, PO-D, PG-D, PSj-D, and PSp-F. The compositions of metabolites were different between fresh and dried mushrooms, especially in *P. sajorcaju* and *H. erinaceus* (Figure 4a,b). Primary metabolites, especially saccharides, lipids, and amino acids, could affect plants’ own growth and involvement in the biosynthesis of necessary materials [37,38]. The content of metabolites from the same genus may have great differences, such as *Agricale* with different species. The metabolites of mushrooms contained abundant sugars. Mannitol, trehalose, and glucose were the main constituents in the tested mushrooms, which coincided with previous studies [39]. Sugars had great effects, not only in cellular energy metabolism, but also on the formation of structural polysaccharides [40,41]. The various alcohol derivatives, especially mannitol and arabinitol transformed from sugars, were reported to support the growth of the fruiting bodies of mushrooms [30]. Except for sugars and amino acids, 2-octenoate and 1-octen-3-ol, which belong to the “fungi alcohol”, were important elements for the special fragrance present in mushrooms [41]. Aspartic and glutanmic acids in mushrooms could give the most typical mushroom taste, umami, or palatable taste [42]. The fresh mushrooms were immensely distinguished from dried ones in compositions and metabolites.

**Figure 5:**
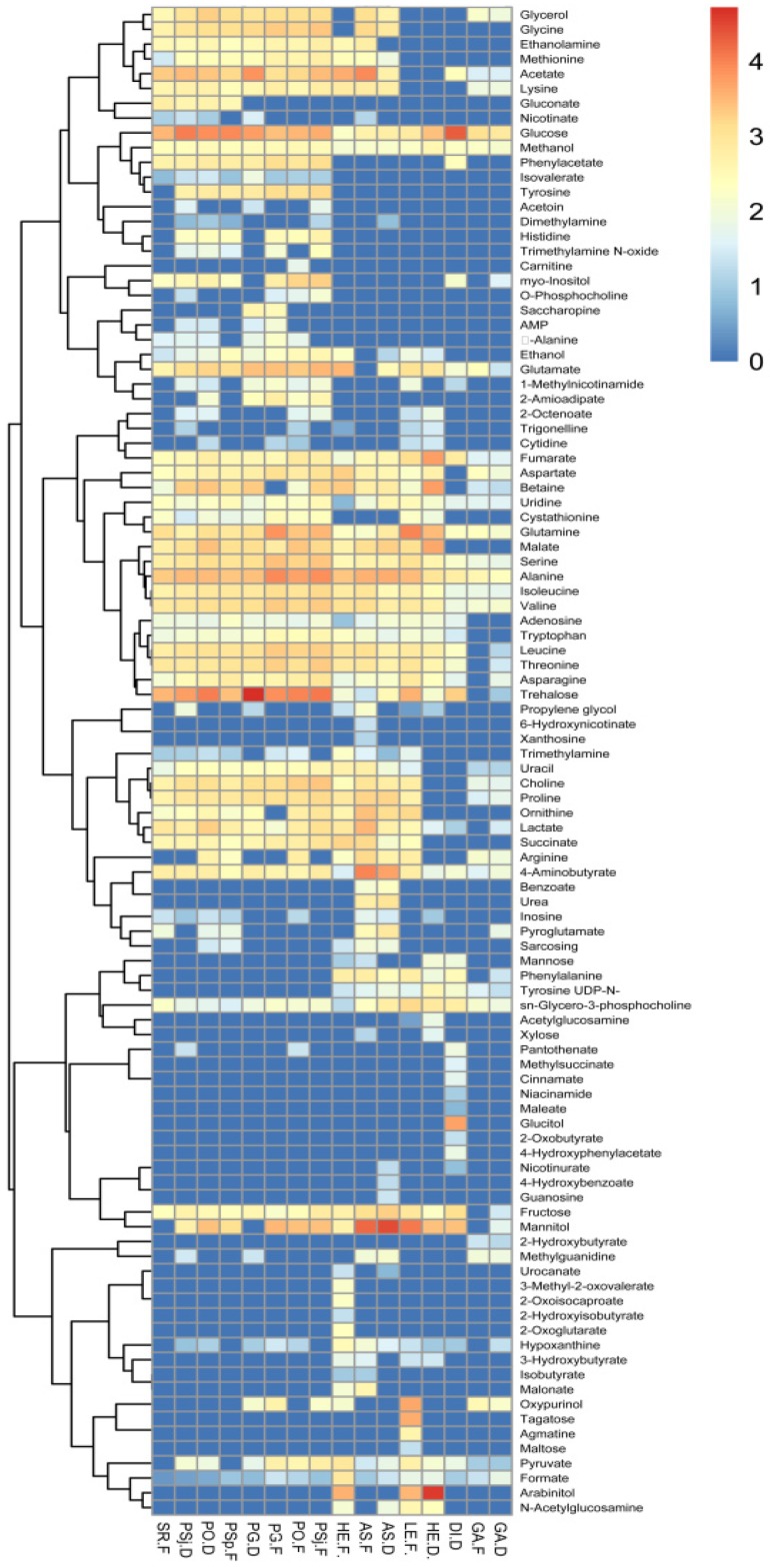
Heatmap of small molecules from the dried and fresh mushrooms. The red color indicates the content more than 2.5 mM, and the blue color indicates the content less than 2.5 mM.

The same results could be obtained by heatmap analysis (Figure 5). HE-F was rich in lysine, acetate, methionine, ethanolamine, trimethylamine, uracia, choline, proline, ornithine, arginine, succinate, sarcosing, urocanate, 3-methyl-2-oxovalerate, 2-hydroxyisovutyrate, 2-oxoglutarate, oxypurinol, malonate, and isobutyrate. In contrast, HE-D had more abundant malate, glutamine, arabinitol, and mannitol. Some compositions of metabolites existed in all mushrooms, but others were only found in one or few mushrooms. The metabolites with glucose, methanol, fumarate, uridine, glutamine, serine, alanine, isoleucine, valine, 4-aminobutyrate, formate, and sn-glycero-3-phosphocholine were found in all selected mushrooms (Figure 5). The metabolites, such as 3-methyl-2-oxovalerate, 2-oxoisocaproate, 2-hydroxyisobutyrate, and 2-oxoglutarate, were only found in HE-F, while 4-hydroxybenzoate and guanosine only existed in AS-D, and pantothenate was only found in PSj-D, PO-F, and DI-D. The metabolites of HE-F were also quite different from HE-D.

**Figure 6:**
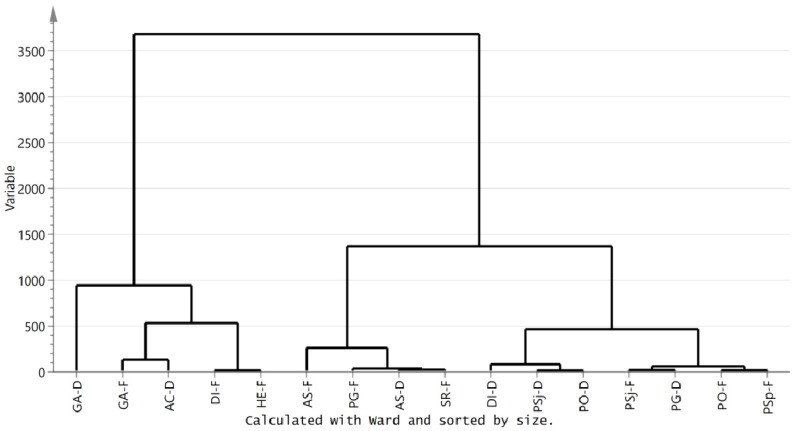
Clustering analysis of compositions from the dried and fresh edible mushrooms.

Clustering analysis of compositions from dried and fresh edible and medical mushrooms showed that they could be divided into three kinds of mushrooms with the distance for 1000 (Figure 6). Cluster I was composed of HE-F, GA-D, GA-F, AC-D, and DI-F. Cluster II was made up of AS-F, AS-D, PG-F, and SR-F. Cluster III contained DI-D, PSj-D, PO-D, PSj-F, PG-D, PO-F, and PSp-F. The metabolic compositions of mushrooms showed great differences, even in the same order. Cluster II and Cluster III were species of *Agaricales*. However, these two clusters had much difference. Interestingly, there was a difference in terms of the mushrooms’ composition. For instance, the dried and fresh *D. indusiata* were divided into two clusters, which may be caused by the big difference between them. Moreover, the fresh *G. amboinense* were similar in composition with dried *A. camphorata.* It was demonstrated that the drying process may influence the composition of mushrooms. The different functions of the mushrooms may be closely associated with the nutrient (protein, fat, carbohydrate, vitamins, trace minerals, and amino acids) composition of mushrooms. These data would be a desirable choice for analyzing the functions of mushrooms.

## 4. Conclusions

Mushrooms are traditionally regarded as nutritional and delicious foods worldwide. Because of their abundant bioactive phytochemicals, they are also generally present in traditional Chinese medicine. In this study, we presented a systematic broad-scale metabolomic investigation of 11 species of dried and fresh edible and medicinal mushrooms. The nutritional component analysis of these selected 11 species suggested that mushrooms contained a wide range of proteins, carbohydrates, amino acids, vitamins, and small molecules. The results showing the chemical components of the selected mushrooms provide fundamental data for the development of functional foods from mushrooms.

## Figures and Tables

**Table 1:** Proximate compositions and trace elements from the dried and fresh edible mushrooms.

Macrofungi	Proximate Composition						Trace Elements (mg/kg)				
	Protein (g/100 g)	Fat (g/100 g)	Carbohydrates (g/100 g)	Coarse Fibers (g/100 g)	Vitamin A (μg/100 g)	Vitamin C (mg/100 g)	Cu	Zn	Fe	Mn	Ca
GA-D	12.7	0.8	45	40.8	15.2	10.5	4.7	19.2	13.4	11	321
GA-F	14.6	1	61	17.7	17.8	1.6	2.2	5.9	3.9	2.2	172
AS-D	37.2	2.2	23.9	0.075	<16	69.7	42.6	28	28.4	1.1	50.2
AS-F	50.4	15.7	18	1.2	7.38	1.1	14.6	13.5	14.4	0.9	53.8
DI-D	19.5	2.7	41.6	0.087	21	111.4	15	130	184	75	468
DI-F	1.9	2.6	40.2	0.085	770	16.7	0.83	0.39	1.7	-	4.1
PSj-D	23.2	2.7	49.4	0.4	1.46	0.6	0.6	4.8	5.4	0.7	14
PSj-F	35.5	1.98	40.1	0.8	4.05	0.6	1.9	9	8.2	1.2	18
PO-D	21.1	2.19	50.7	0.7	6.51	0.7	3.6	6.8	15	0.8	7
PO-F	36.4	2.06	34.7	0.5	4.42	0.5	2.5	7.7	14	1	11
PG-D	32.7	1.71	42.8	0.9	21.9	0.6	28	15	25	1.1	24
PG-F	39.8	2.26	29	0.8	2.98	0.8	6.6	13	15	1.4	22
HE-F	2.3	1.8	42.9	0.42	580	5.5	6.1	10.4	0.15	0.079	39
SR-F	41.1	4.5	22.4	0.4	1.5	1.6	2.1	3.4	13	1.4	32
PSp-F	33.8	4.1	34.6	0.5	2.61	0.5	1.2	8.4	10	1.2	8.5
AC-D	4.1	3	52.8	12.2	-	0.6	3.2	27	38	12	740

Note: GA-D, *G. amboinense* (dried); GA-F, *G. amboinense* (fresh); AS-D, *A. subrufescens* (dried); AS-F, *A. subrufescens* (fresh); DI-D, *D. indusiata* (dried); DI-F, *D. indusiata* (fresh); PSj-D, *P. sajorcaju* (dried); PSj-F, *P. sajorcaju* (fresh); PO-D, *P. ostreatus* (dried); PO-F, *P. ostreatus* (fresh); PG-D, *P. geesteranu* (dried); PG-F, *P.geesteranu* (fresh); HE-F, *H. erinaceus* (fresh); SR-F: *S. rugosoannulata* (fresh); PSp-F, *P. sapidus* (fresh); AC-D, *A. camphorata* (dried). Protein and fat were calculated by dried basis. “-“: not detected.

**Table 2:** The amino acid composition of the dried and fresh edible mushrooms (dried weight: g/kg).

Amino Acid	GA-D	GA-F	AS-F	DI-F	PSj-D	PSj-F	PO-D	PO-F	PG-D	PG-F	HE-F	SR-F	PSp-F	AC-D	LE-F
TEAA	60.5	72	133.7	9.9	7.5	13.8	7.5	13.3	15.3	17.4	36.8	7.3	6.7	6	107.7
TNAA	23.5	30	91.2	6.4	6.5	10.3	6	10.6	12.4	14	18.1	5.4	4.3	7.4	110.1
CEAA	1.4	2.8	5.9	1.9	1.5	1.2	0.6	1.2	1.3	1.5	2.7	0.4	0.9	0.8	4.8
Asp	5.8	8.1	17.7	1.4	0.9	2.2	1.2	2.1	2.7	3.1	5.3	0.9	0.6	1.9	16.8
Thr	3.6	4.8	11	0.9	0.8	1.5	0.8	1.4	1.7	1.9	3.2	0.8	0.4	1.2	10.3
Met	33.2	36.3	33.5	0.7	0.4	0.9	0.5	0.8	0.8	0.8	11.6	0.2	0.2	-	29.7
Ile	2.7	3.4	9.8	0.7	0.7	1.3	0.6	1.2	1.5	1.6	2	0.6	0.8	1.1	7.1
Leu	4.2	5.3	16.8	1.2	1.1	2	1	1.9	2.1	2.6	3.8	1.3	1.4	1.6	11.7
Val	3.5	4.7	11.9	2.7	1.6	2.3	1.6	2.4	2.4	2.7	2.9	1.6	1.8	-	9.6
Phe	2.7	3.8	10.9	0.9	0.7	1	0.6	1	1.3	1.3	2.3	0.6	0.8	0.9	8.5
Lys	3.5	3.9	16.6	1	0.9	1.9	0.9	1.8	2	2.5	4.1	0.9	0.3	0.8	10.2
His	1.3	1.7	5.5	0.4	0.4	0.7	0.3	0.7	0.8	0.9	1.6	0.4	0.4	0.4	3.8
Ser	3.9	4.7	8.8	0.9	0.6	1.4	0.8	1.3	1.5	1.7	3	0.7	0.1	1.3	10.8
Glu	6.6	8.6	21.6	2.4	2.5	3.1	2.1	3.5	4.9	5.1	1.8	1.8	1.5	2.1	60.8
Gly	3.4	4.3	11.6	0.7	0.8	1.3	0.7	1.2	1.6	1.8	2.6	0.6	0.9	1.2	8.9
Ala	4	5.3	16	1.1	1.4	2.7	1.3	2.5	2.8	3.1	3.1	1.1	1	1.3	12.2
Arg	2.7	3.6	20.9	0.7	0.1	0.5	0.2	0.9	0.3	0.6	5.2	0.3	0.2	0.4	10.6
Pro	2.9	3.5	12.3	0.6	1.1	1.3	0.9	1.2	1.3	1.7	2.4	0.9	0.6	1.1	6.8
Cys	0.1	1	1.5	1.1	0.8	-	-	-	-	-	0.7	-	-	-	1.9
Tyr	1.3	1.8	4.4	0.8	0.7	1.2	0.6	1.2	1.3	1.5	2	0.4	0.9	0.8	2.9

Note: GA-D, *G. amboinense* (dried); GA-F, *G. amboinense* (fresh); AS-D, *A. subrufescens* (dried); AS-F, *A. subrufescens* (fresh); DI-D, *D. indusiata* (dried); DI-F, *D. indusiata* (fresh); PSj-D, *P. sajorcaju* (dried); PSj-F, *P. sajorcaju* (fresh); PO-D, *P. ostreatus* (dried); PO-F, *P. ostreatus* (fresh); PG-D, *P. geesteranu* (dried); PG-F, *P. geesteranu* (fresh); HE-F, *H. erinaceus* (fresh); SR-F: *S. rugosoannulata* (fresh); PS-F, *P. sapidus* (fresh); AC-D, *A. camphorata* (dried); LE-F, *L. edodes* (Berk.) Sing (fresh). TEAA: total essential amino acids; TNAA: total non-essential amino acids; CEAA: conditionally essential amino acids; “-“: not detected.

## Supplementary Materials

The following are available online at https://www.mdpi.com/2304-8158/8/9/397/s1, Table S1: The concentrations of small molecules from dried and fresh edible mushrooms measured by nuclear magnetic resonance spectroscopy. Figure S1: The nuclear magnetic resonance (NMR) spectra of mushrooms.
