# Supplementary material for: Nutrient Properties and Nuclear Magnetic Resonance-Based Metabonomic Analysis of Macrofungi
Source: Foods. 2019 Sep 7;8(9):397. doi: 10.3390/foods8090397 (PMC6769546; doi:10.3390/foods8090397)
Supplement: Supplementary file 1 [file foods-08-00397-s001.pdf]

Supplementary Table S1 The concentrations of small molecules from dried and fresh edible mushrooms measured by nuclear magnetic resonance spectroscopy.

	Dried and fresh edible mushrooms (mmol/L)															
	LE-F	HE-D	HE-F	AS-F	AS-D	GA-F	GA-D	DI-D	PO-D	PO-F	PSj--D	PSj-F	PSp-F	SR-F	PF-D	PG-F
Pantothenate (C1)	-	-	-	-	-	-	-	0.0801	-	0.0237	0.02	-	-	-	-	-
1-Methylnicotinamide (C2)	0.0874	-	-	-	-	-	-	0.0153	0.026	0.0534	0.0494	0.1103	-	-	0.0959	0.1485
2-Amioadipate (C3)	-	-	-	-	-	-	-	-	0.1303	0.1706	-	0.3864	-	-	0.2452	0.6474
2-Hydroxybutyrate (C4)	-	-	-	-	-	0.0231	0.0145	-	-	-	-	-	-	-	-	-
2-Hydroxyisobutyrate (C5)	-	-	0.0181	-	-	-	-	-	-	-	-	-	-	-	-	-
2-Octenoate (C6)	0.0203	0.0747	-	-	-	-	-	-	0.04	0.0475	0.0384	0.0742	-	-	-	-
2-Oxobutyrate (C7)	-	-	-	-	-	-	-	0.0196	-	-	-	-	-	-	-	-
2-Oxoglutarate (C8)	-	-	0.2156	-	-	-	-	-	-	-	-	-	-	-	-	-
2-Oxoisocaproate (C9)	-	-	0.1805	-	-	-	-	-	-	-	-	-	-	-	-	-
3-Hydroxybutyrate (C10)	0.0226	0.0264	0.0651	0.0436	-	-	-	-	-	-	-	-	-	-	-	-
3-Methyl-2-oxovalerate (C11)	-	-	0.1628	-	-	-	-	-	-	-	-	-	-	-	-	-
4-Aminobutyrate (C12)	0.6329	0.0641	0.034	9.4652	5.0904	0.0423	0.1031	0.1192	0.5624	0.3589	0.6458	0.6915	0.261	0.6378	0.6365	0.4054
4-Hydroxybenzoate (C13)	-	-	-	-	0.016	-	-	-	-	-	-	-	-	-	-	-
4-Hydroxyphenylacetate (C14)	-	-	-	-	-	-	-	0.0701	-	-	-	-	-	-	-	-
6-Hydroxynicotinate (C15)	-	-	-	0.0214	-	-	-	-	-	-	-	-	-	-	-	-
Acetate (C16)	-	-	4.0214	8.9391	0.4969	0.0331	0.0351	0.2325	2.3635	1.5927	3.0555	2.9952	1.5678	2.1075	7.4077	1.0747
Acetoin (C17)	-	-	-	-	-	-	-	-	-	-	0.0426	0.0558	-	-	0.0238	-
Acetylglucosamine (C18)	0.0023	0.0742	-	-	-	-	-	-	-	-	-	-	-	-	-	-
Adenosine (C19)	0.1427	0.109	0.0068	0.053	0.1296	-	-	0.0497	0.0679	0.0602	0.0801	0.1159	0.1686	0.1002	0.0803	0.0819
Agmatine (C20)	0.4252	-	-	-	-	-	-	-	-	-	-	-	-	-	-	-
Alanine (C21)	2.8888	1.0392	2.4675	3.6525	4.0048	0.3773	0.2547	0.6151	3.066	4.9977	2.8019	7.4748	2.3505	2.1253	2.6378	9.1295
AMP (C22)	-	-	-	-	-	-	-	-	0.0259	-	0.029	-	-	-	0.0344	0.117
Arabinitol (C23)	3.3856	41.3413	3.7722	-	-	-	-	-	-	-	-	-	-	-	-	-
Arginine (C24)	0.6601	-	0.1712	1.5424	0.4882	0.1436	0.0899	-	0.4577	0.6653	-	-	0.2039	-	-	-
Asparagine (C25)	0.787	0.2158	0.0718	0.161	0.1477	-	0.0614	0.0531	0.4773	0.5787	0.2852	1.0443	0.7234	0.1201	0.3478	0.3954
Aspartate (C26)	0.1705	0.5386	1.9756	0.4534	0.3645	0.2077	0.0959	-	0.5295	0.7527	0.3863	1.134	0.4542	0.2165	0.5687	1.222
Benzoate (C27)	-	-	-	0.1261	0.1981	-	-	-	-	-	-	-	-	-	-	-
Betaine (C28)	0.1414	5.8416	2.1201	0.5655	0.6987	0.0252	0.0163	-	2.3414	0.1199	1.9836	1.7987	1.2359	0.1001	2.1453	-
Carnitine (C29)	-	-	-	-	-	-	-	-	-	0.0588	-	-	-	-	-	-
Choline (C30)	0.6286	-	0.2809	1.0201	0.6144	0.063	0.0535	-	1.2262	1.8892	1.1535	2.3695	0.6582	0.459	1.1794	1.3062
Cinnamate (C31)	-	-	-	-	-	-	-	0.0501	-	-	-	-	-	-	-	-
Cystathionine (C32)	0.1827	0.0906	-	-	-	-	-	-	0.1149	0.1895	0.0284	0.233	0.0643	0.1725	0.0822	0.3057
Cytidine (C33)	0.0183	0.0262	-	-	-	-	-	-	0.0167	0.0086	-	-	-	-	-	0.0141
Dimethylamine (C34)	-	-	-	-	0.006	-	-	-	0.0082	0	0.0053	0.0136	0.0041	-	-	-
Ethanol (C35)	0.0843	0.0289	0.1727	-	0.0131	-	-	-	0.0818	0.3157	0.0589	0.386	0.2775	0.0249	0.0954	0.1927
Ethanolamine (C36)	-	-	0.3186	0.5893	-	-	-	-	0.3562	0.3788	0.3069	0.5782	0.2204	0.3235	0.3387	0.3988
Formate (C37)	0.0681	0.0618	0.7621	0.0079	0.0248	0.0206	0.0672	0.0092	0.003	0.0127	0.0023	0.0065	0.0077	0.0015	0.0048	0.0228
Fructose (C38)	0.9121	0.1912	0.5182	1.2196	1.9429	-	0.027	1.229	0.3194	0.2786	0.4425	0.7047	0.2379	0.2308	0.3888	0.1595
Fumarate (C39)	1.3787	5.7174	0.098	0.3235	0.3729	0.042	0.0417	0.7324	0.6736	0.7984	0.3301	0.6528	0.366	0.2605	0.3848	0.3294
Glucitol (C40)	-	-	-	-	-	-	-	5.2746	-	-	-	-	-	-	-	-
Gluconate (C41)	-	-	-	-	-	-	-	-	0.4579	-	0.4241	-	0.3482	0.677	-	-
Glucose (C42)	0.7487	2.5954	0.1676	0.4784	0.5687	1.2314	0.767	22.0244	8.2627	3.4077	11.0376	4.1885	8.7704	3.341	5.754	2.6426
Glutamate (C43)	1.0592	0.8507	3.3497	0	0.3168	0.234	0.0234	0.129	1.7061	2.2255	1.1977	3.2714	1.3506	0.4293	2.6796	2.7372
Glutamine (C44)	10.0812	2.8578	0.1836	0.1553	0.6846	0.2185	0.1415	0.1858	1.2347	2.3087	0.4573	3.426	0.8738	1.4093	1.2132	6.8549

Glycerol (C45)	-	-	-	1.2614	0.4396	0.1532	0.0944	-	1.8968	1.261	0.9601	1.3143	1.1859	0.3349	1.0137	1.2228
Glycine (C46)	-	-	-	1.6136	0.7865	-	-	-	1.2322	1.6593	1.0334	2.4376	1.2738	0.4789	1.4792	2.0624
Guanosine (C47)	-	-	-	-	0.0236	-	-	-	-	-	-	-	-	-	-	-
Histidine (C48)	-	-	-	-	-	-	-	-	0.281	0.2689	0.1853	0.4736	0.2512	-	-	0.3088
Hypoxanthine (C49)	0.0216	0.0084	0.2892	0.1145	0.0354	-	0.0194	0.0084	0.0106	0.0135	0.0065	-	-	-	0.0099	0.0267
Inosine (C50)	-	0.0085	-	0.0511	0.0289	-	-	-	0.0211	0.0158	0.0077	-	0.0133	0.0207	-	-
Isobutyrate (C51)	-	-	0.0078	0.0089	-	-	-	-	-	-	-	-	-	-	-	-
Isoleucine (C52)	0.5426	0.2803	0.7435	0.9817	0.3293	0.0709	0.0595	0.071	0.8664	1.0291	0.7095	1.5767	0.9201	0.4889	0.7585	1.3844
Isovalerate (C53)	-	-	-	-	-	-	-	-	0.0251	0.0106	0.021	0.0108	0.0068	0.0048	0.0778	0.0087
Lactate (C54)	0.3324	0.0396	0.8413	3.4312	0.4903	-	0.0284	0.0103	1.8599	0.6198	0.6738	1.2706	0.5652	0.882	0.3369	0.1265
Leucine (C55)	0.8113	0.6019	1.0349	1.2634	0.4933	-	0.0128	0.1962	1.1455	1.748	0.9453	1.9518	1.0499	0.6578	1.0524	2.1146
Lysine (C56)	-	-	0.6867	0.6967	0.5669	0.0822	0.0803	-	0.5058	0.3371	0.4945	0.6607	0.2736	0.3983	0.3612	0.6459
Malate (C57)	1.1778	4.3212	0.4895	1.3579	1.8462	-	-	-	2.5558	2.4234	1.1158	1.5901	1.2449	0.5948	1.3063	0.719
Maleate (C58)	-	-	-	-	-	-	-	0.0042	-	-	-	-	-	-	-	-
Malonate (C59)	-	-	0.1208	0.3934	-	-	-	-	-	-	-	-	-	-	-	-
Maltose (C60)	0.0197	-	-	-	-	-	-	-	-	-	-	-	-	-	-	-
Mannitol (C61)	12.2358	2.6523	0.4895	17.7227	28.57	-	0.0517	2.546	2.7563	2.566	0.5063	2.7812	1.2496	-	-	3.3323
Mannose (C62)	0	0.1152	0.0097	0.02	-	-	-	0.0877	-	-	-	-	-	-	-	-
Methanol (C63)	0.1681	0.3548	0.1289	0.1623	0.1584	0.1655	0.1444	0.1853	0.3119	0.3373	0.2677	0.2965	0.2693	0.2685	0.2848	0.2984
Methionine (C64)	-	-	0.2642	0.2307	0.0807	-	-	-	0.2715	0.4009	0.2301	0.5494	0.2583	0.028	0.2783	0.4664
Methylguanidine (C65)	-	-	-	0.1058	0.1433	0.0991	0.0789	-	-	-	0.0252	-	-	-	0.0246	-
Methylsuccinate (C66)	-	-	-	-	-	-	-	0.0363	-	-	-	-	-	-	-	-
myo-lnositol (C67)	-	-	-	-	-	-	0.0365	0.1346	0.461	1.7525	0.3497	1.8282	0.1864	0.1841	-	0.5536
N-Acetylglucosamine (C68)	0.3891	0.2436	0.1191	-	0.0838	-	-	-	-	-	-	-	-	-	-	-
Niacinamide (C69)	-	-	-	-	-	-	-	0.0097	-	-	-	-	-	-	-	-
Nicotinate (C70)	-	-	-	-	-	-	-	-	0.0099	-	0.02	-	-	0.0107	0.0328	-
Nicotinate (C71)	-	-	-	0.0129	-	-	-	-	-	-	-	-	-	-	-	-
Nicotinurate (C72)	-	-	-	-	0.0159	-	-	0.0054	-	-	-	-	-	-	-	-
O-Phosphocholine (C73)	-	-	-	-	-	-	-	-	-	0.0531	0.0193	0.1229	-	-	-	0.0349
Ornithine (C74)	1.5895	-	0.3951	2.5903	1.3205	-	-	-	0.361	0.5585	0.2143	0.9855	0.1594	0.1572	0.2824	-
Oxypurinol (C75)	4.3608	-	0.1444	-	-	0.3527	0.1529	-	-	-	-	0.1608	-	-	0.1438	0.4802
Phenylacetate (C76)	-	-	-	-	-	-	-	0.2414	0.6993	0.9089	0.5396	1.3577	0.6793	0.4661	0.6624	1.4456
Phenylalanine (C77)	0.6666	0.0863	0.6044	0.6684	0.2833	-	0.025	0.3082								
Proline (C78)	0.4594	-	0.778	1.6283	1.7706	0.0346	0.0571	-	0.9338	1.165	0.8475	1.5268	0.7923	0.4642	1.1011	1.15
Propylene glycol (C79)	0.0018	0.0097	0.0214	0.1638	-	-	-	-	-	-	0.0917	-	-	-	0.0152	-
Pyroglutamate (C80)	-	-	-	0.2885	0.8026	-	0.0637	-	0.055	-	-	-	0.0719	0.0866	-	-
Pyruvate (C81)	0.5257	0.1274	0.8735	0.0272	0.0631	0.0091	0.0086	0.075	0.0827	0.3889	0.1226	0.5983	-	-	0.0576	0.5073
Saccharopine (C82)	-	-	-	-	-	-	-	-	-	-	-	-	-	-	0.4274	0.2996
Sarcosing (C83)	-	-	0.0237	0.1097	0.0816	-	-	-	0.0255	-	-	-	0.0407	-	-	-
Serine (C84)	1.1382	0.4335	0.3224	0.4485	0.4644	0.0843	0.0677	0.1538	1.3591	1.645	0.9001	2.4169	0.995	0.6016	1.2704	2.5491
sn-Glycero-3-phosphocholine (C85)	1.4901	0.7781	0.0148	0.1918	0.6659	0.1462	0.0819	0.6716	0.0526	0.1311	0.0648	0.1431	0.0325	0.1785	0.0787	0.1539
Succinate (C86)	0.248	-	1.746	1.5501	0.1448	-	-	-	0.4147	0.5667	0.3993	1.0218	0.2048	0.3832	0.3535	0.6049
Tagatose (C87)	4.1042	-	-	-	-	-	-	-	-	-	-	-	-	-	-	-
Threonine (C88)	1.0427	0.4303	0.795	0.9364	0.462	-	0.0257	0.1424	0.916	1.1432	0.8391	2.048	1.0035	0.8703	1.103	1.8575
Trehalose (C89)	3.5988	0.14	0.1126	0.0227	0.3257	-	0.0079	1.8266	10.5894	9.8608	5.4959	12.2749	2.5185	3.4123	53	8.1527
Trigonelline (C90)	0.0142	0.03	0.0029	-	-	-	-	-	-	0.0123	0.0116	-	-	-	-	-
Trimethylamine (C91)	0.0492	-	0.1739	0.0371	0.0049	-	-	-	0.0199	0.0368	0.0104	-	0.0104	0.0099	-	0.0266

Trimethylamine N-oxide (C92)	-	-	-	-	-	-	-	-	0.0766	-	0.052	0.2374	0.0373	-	-	0.1238
Tryptophan (C93)	0.1201	0.0959	0.1954	0.1364	0.0598	-	-	0.0286	0.1447	0.2616	0.1244	0.3889	0.1168	0.0835	0.1581	0.3229
Tyrosine (C94)	-	-	-	-	-	-	-	-	0.7482	1.0553	0.4911	1.4723	0.7155	-	0.7372	1.3831
Tyrosine UDP-N- (C95)	0.0458	0.3819	0.023	0.0473	0.0834	0.038	0.0193	0.1369	-	-	-	-	-	-	-	-
Uracil (C96)	0.0433	-	0.5384	0.5643	0.0957	0.0134	0.012	-	0.2509	0.2634	0.2274	0.3537	0.1856	0.0703	0.2762	0.2727
Urea (C97)	-	-	-	0.5576	1.0054	-	-	-	-	-	-	-	-	-	-	-
Uridine (C98)	0.3049	0.1357	0.0044	0.0968	0.3623	0.0449	0.0423	0.0513	0.1973	0.1756	0.1241	0.3422	0.2959	0.217	0.0857	0.1964
Urocanate (C99)	-	-	0.0218	-	0.0046	-	-	-	-	-	-	-	-	-	-	-
Valine (C100)	0.8434	0.503	1.1693	1.3238	0.5691	0.1259	0.1412	0.078	1.2236	1.4776	0.9879	2.1755	1.2591	0.7822	1.2418	2.1819
Xanthosine (C101)	-	-	-	0.0132	-	-	-	-	-	-	-	-	-	-	-	-
Xylose (C102)	0	0.0442	0	0.014	-	-	-	-	-	-	-	-	-	-	-	-
β-Alanine (C103)	-	-	-	-	-	-	-	-	0.0374	0.0577	0.0474	-	-	0.039	0.0635	0.1693
Sum	60.8738	73.5667	34.3196	69.6617	58.8409	4.0818	3.1038	37.8981	56.9478	55.4982	44.0248	76.0795	38.0112	24.9111	94.0427	63.3686

Note: GA-D, *G. amboinense* (dried); GA-F, *G. amboinense* (Fresh); AS-D, *A. subrufescents* (dried); AS-F, *A. subrufescents* (Fresh); DI-D, *D. indusiata* (dried); DI-F, *D. indusiata* (Fresh); PSj-D, *P. sajorcaju* (dried); PSj-F, *P. sajorcaju* (Fresh); PO-D, *P. ostreatus* (dried); PO-F, *P. ostreatus* (Fresh); PG-D, *P. geesteranu* (dried); PG-F, *P.geesteranu* (Fresh); HE-D, *H. erinaceus* (dried); HE-F, *H. erinaceus* (Fresh); SR-F: *S. rugosoannulata* (Fresh); PSp-F, *P. sapidus* (Fresh); AC-D, *A. camphorata* (dried). "-": not detected.

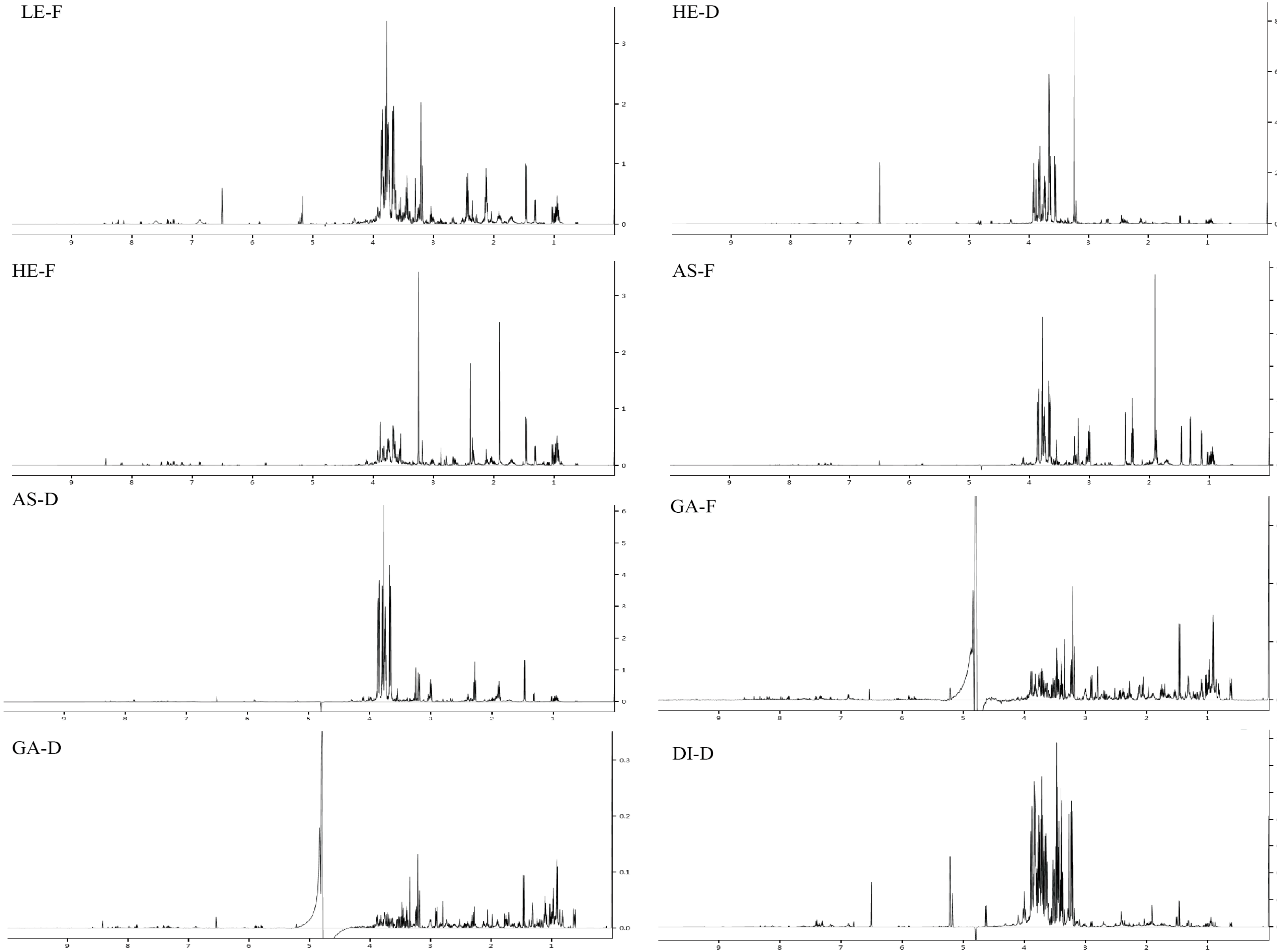
LE-F
HE-D
HE-F
AS-F
AS-D
GA-F
GA-D
DI-D

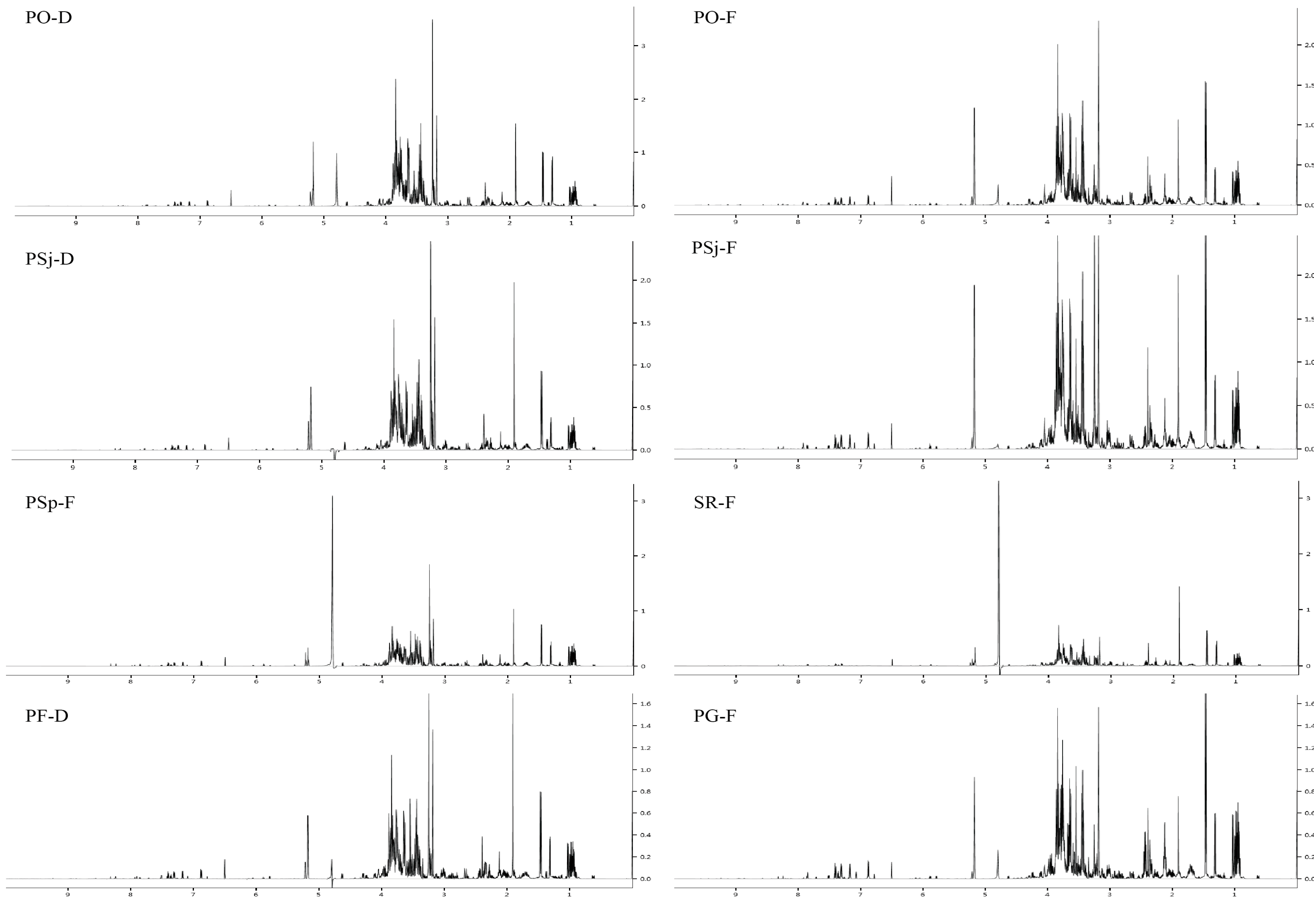
PO-D
PO-F
PSj-D
PSj-F
PSp-F
SR-F
PF-D
PG-F

Supplementary Figure S1. The NMR spectra of mushrooms. GA-D, *G. amboinense* (dried); GA-F, *G. amboinense* (Fresh); AS-D, *A. subrufescents* (dried); AS-F, *A. subrufescents* (Fresh); DI-D, *D. indusiata* (dried); DI-F, *D. indusiata* (Fresh); PSj-D, *P. sajorcaju* (dried); PSj-F, *P. sajorcaju* (Fresh); PO-D, *P. ostreatus* (dried); PO-F, *P. ostreatus* (Fresh); PG-D, *P. geesteranu* (dried); PG-F, *P.geesteranu* (Fresh); HE-D, *H. erinaceus* (dried); HE-F, *H. erinaceus* (Fresh); SR-F: *S. rugosoannulata* (Fresh); PSp-F, *P. sapidus* (Fresh); AC-D, *A. camphorata* (dried).
